# miR393 Is Required for Production of Proper Auxin Signalling Outputs

**DOI:** 10.1371/journal.pone.0095972

**Published:** 2014-04-24

**Authors:** David Windels, Dawid Bielewicz, Miryam Ebneter, Artur Jarmolowski, Zofia Szweykowska-Kulinska, Franck Vazquez

**Affiliations:** 1 Department of Environmental Sciences, Section of Plant Physiology, University of Basel, Zurich-Basel Plant Science Center, Part of the Swiss Plant Science Web, Basel, Switzerland; 2 Department of Gene Expression, Faculty of Biology, Adam Mickiewicz University, Poznan, Poland; Iwate University, Japan

## Abstract

Auxins are crucial for plant growth and development. Auxin signalling primarily depends on four partially redundant F-box proteins of the TIR1/AFB2 Auxin Receptor (TAAR) clade to trigger the degradation of AUX/IAA transcriptional repressors. Auxin signalling is a balanced system which involves complex feedback regulations. miR393 regulation of *TAAR* genes is important for different developmental programs and for responses to environment. However, so far, the relevance of the two *MIR393* genes for Arabidopsis leaf development and their significance for auxin signalling homeostasis have not been evaluated. First, our analyses of *mir393a-1* and *mir393b-1* mutants and of *mir393ab* double mutant show that the two genes have only partially redundant functions for leaf development. Expression analyses of typical auxin-induced reporter genes have shown that the loss of miR393 lead to several unanticipated changes in auxin signalling. The expression of *DR5pro:GUS* is decreased, the expression of primary *AUX/IAA* auxin-responsive genes is slightly increased and the degradation of the AXR3-NT:GUS reporter protein is delayed in *mir393ab* mutants. Additional analyses using synthetic auxin and auxin antagonists indicated that miR393 deficient mutants have higher levels of endogenous AUX/IAA proteins, which in turn create a competition for degradation. We propose that the counter-intuitive changes in the expression of *AUX/IAA* genes and in the accumulation of AUX/IAA proteins are explained by the intrinsic nature of *AUX/IAA* genes which are feedback regulated by the AUX/IAA proteins which they produce. Altogether our experiments provide an additional highlight of the complexity of auxin signaling homeostasis and show that miR393 is an important component of this homeostasis.

## Introduction

Auxins are phytohormones important for plant growth, organogenesis and various responses to environmental changes [1],[2]. Auxin signalling primarily depends on perception by four partially-redundant auxin receptors of the TRANSPORT INHIBITOR RESPONSE 1 (TIR1)/AUXIN SIGNALLING F-BOX PROTEIN 2 (AFB2) clade [3]–[5]. Upon binding auxins, these TAAR proteins, which are the specificity-components of SKIP/CULLIN/F-BOX (SCF)-ubiquitin ligase complexes, form a co-receptor complex with AUXIN/INDOLE-3-ACETIC ACID (AUX/IAA) transcriptional repressors [6]–[8]. AUX/IAA proteins are then ubiquitinated and degraded by the 26S proteasome [7]. This leads to the release of AUXIN RESPONSE FACTOR (ARF) transcription factors to which AUX/IAA were bound and allows ARFs to activate or repress the transcription of primary auxin-responsive genes [9]. The homeostasis of auxin signaling involves feedback-regulation of AUX/IAA genes' expression by the AUX/IAA proteins which they generate. The homeostasis of *TAAR* genes expression was shown to involve the microRNA miR393. This regulation is important for innate immunity [10], [11], for root response to nitrate, salinity and drought resistance [12]–[15] and for several aspects of rice and Arabidopsis development [15]–[19]. Our own work has shown that this regulation additionally involves the function of secondary siRNAs, the siTAARs, which are generated from *TAAR* transcripts downstream of the miR393 cleavage site [16], [17]. MiR393 is generated from two distinct genes, *MIR393A* and *MIR393B* [20]. *AtMIR393A*, but not *AtMIR393B*, was shown to be induced by stress while *MIR393B*, but not *AtMIR393A*, was shown to be induced by auxin [10], [19]. Moreover, our work has suggested that miR393 is primarily produced from *AtMIR393A* in the roots and primarily from *AtMIR393B*, in the aerial parts of plants grown in normal growth conditions [17]. Thus, these observations suggested that *MIR393* genes have major distinct functions: *AtMI393A* being primarily involved in the plant responses to environment, and, *AtMIR393B* being primarily involved in the regulation of auxin homeostasis and of auxin-dependent plant development [10]–[19]. Intriguingly, *mir393b-1* mutants which accumulate only trace amounts of miR393 in aerial parts of plants exhibit rather mild phenotypes in normal growth conditions essentially characterized by a pronounced leaf epinasty [17]. Thus, this raised the hypothesis that *AtMIR393A* or other pathways compensate for the loss of *AtMIR393B*.

MiR393 is important for the regulation of *TAAR* genes and is part of a complex homeostatic process which involves feedback transcriptional regulations [19]. However, the significance of miR393 for auxin signalling homeostasis has not been evaluated directly in mutants lacking miR393. To gain insights into these important aspects we have obtained single and double mutants of *MIR393* genes and we have analyzed the impact of these mutations on plant development, on physiological response to auxin and at the molecular level of auxin signalling. The data which we obtained show that the two *MIR393* genes have partially redundant functions for leaf polarity with a primarily role for *AtMIR393B*. Moreover, we have observed that the expression level of the artificial reporter gene *DR5pro:GUS* is slightly decreased in these mutants compared to wt plants while the expression of primary auxin-induced genes is increased. Moreover, experiments using synthetic auxin, auxin antagonists and the *HSpro:AXR3-NT:GUS* reporter gene to monitor the degradation of AUX/IAA proteins showed that the degradation rate of the AXR3-NT:GUS protein is longer in the mutants than in wt plants. These unanticipated results demonstrate that the loss of miR393 leads to complex changes and to simultaneously increase the basal expression level of *AUX/IAA* genes and the basal level of AUX/IAA protein accumulation. Together our data show that miR393 is an important component of the auxin signalling homeostasis required for the establishment of proper and timely auxin signalling outputs.

## Results

### *MIR393* Genes Have Distinct, but Partially Overlapping Expression Patterns

**Figure 1:**
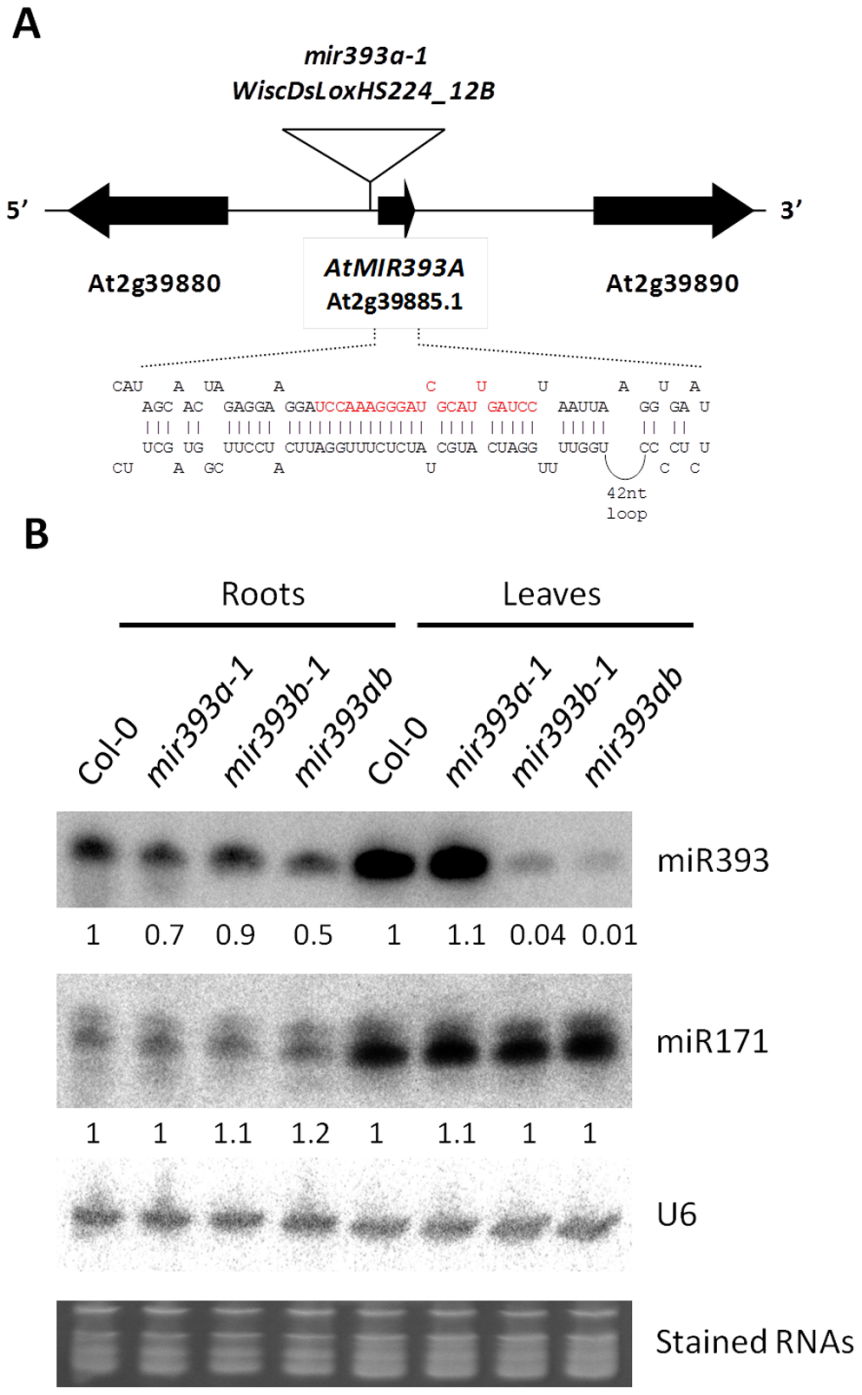
Identification and characterization of *MIR393A* T-DNA insertion mutants. (A) Schematic map showing the 5′-3′ orientation of genes (large arrows and gene names are indicated) flanking *AtMIR393A* on chromosome II. The arrow representing *AtMIR393A* indicates the pri-miRNAs which full-length sequence determined by RACE experiments (given in Fig. S1). The expanded region represents the folded pre-miR393 nucleotide sequence with the miR393 sequence indicated in red. The open triangle represents the T-DNA insertion. The scheme is not drawn to scale. (B) RNA-blot hybridization of RNA prepared from roots and leaves of 34d-old plants. Probed RNAs are indicated on the right. The signal detected for mutants relative to wild-type Col-0 are normalized relative to the *Midori Green* stained RNA signals. Similar results were obtained in three independent experiments.

The microRNA miR393 is encoded by two genes, *AtMIR393A* (*At2g39885*) and *AtMIR393B* (*At3g55734*) (Fig.1A) [21]. Our earlier studies using *mir393b-1* mutants showed that miR393, which primarily arises from *AtMIR393B* in aerial organs, is important for leaf morphology [17]. However, *mir393b-1* mutants exhibit only mild developmental phenotypes. To test whether the weak amounts of miR393 generated from *AtMIR393A* in *miR393b-1* are responsible for this observation, we identified a mutant in the SALK collection which has a T-DNA insertion located in the proximal region of the *AtMIR393A* promoter. Sequencing of the PCR fragments showed that the insertion is located 74-nt upstream of the transcription start which we have identified by Rapid Amplification of cDNA Ends (RACE) experiments (Fig. 1A and S1). In normal growth conditions, this mutant, which we named *mir393a-1*, accumulated 30% lower levels of miR393 in the roots than the wt plants and accumulated normal levels of miR393 in the leaves (Fig.1B). The double mutant *mir393a-1 mir393b-1* (hereafter noted *mir393ab*) accumulated 50% lower levels of miR393 than the wt plant in the roots and only 1% of the wt level in the leaves (Fig. 1B). These experiments showed that *mir393a-1* is not a null-mutant and that the double mutant accumulates lower levels of miR393 than *mir393a-1* or *mir393b-1* alone. These experiments also showed that the two *MIR393* genes have distinct, but partially overlapping expression profiles.

### *MIR393* Genes Have Overlapping, Partially Redundant Functions in Leaf Development

**Figure 2:**
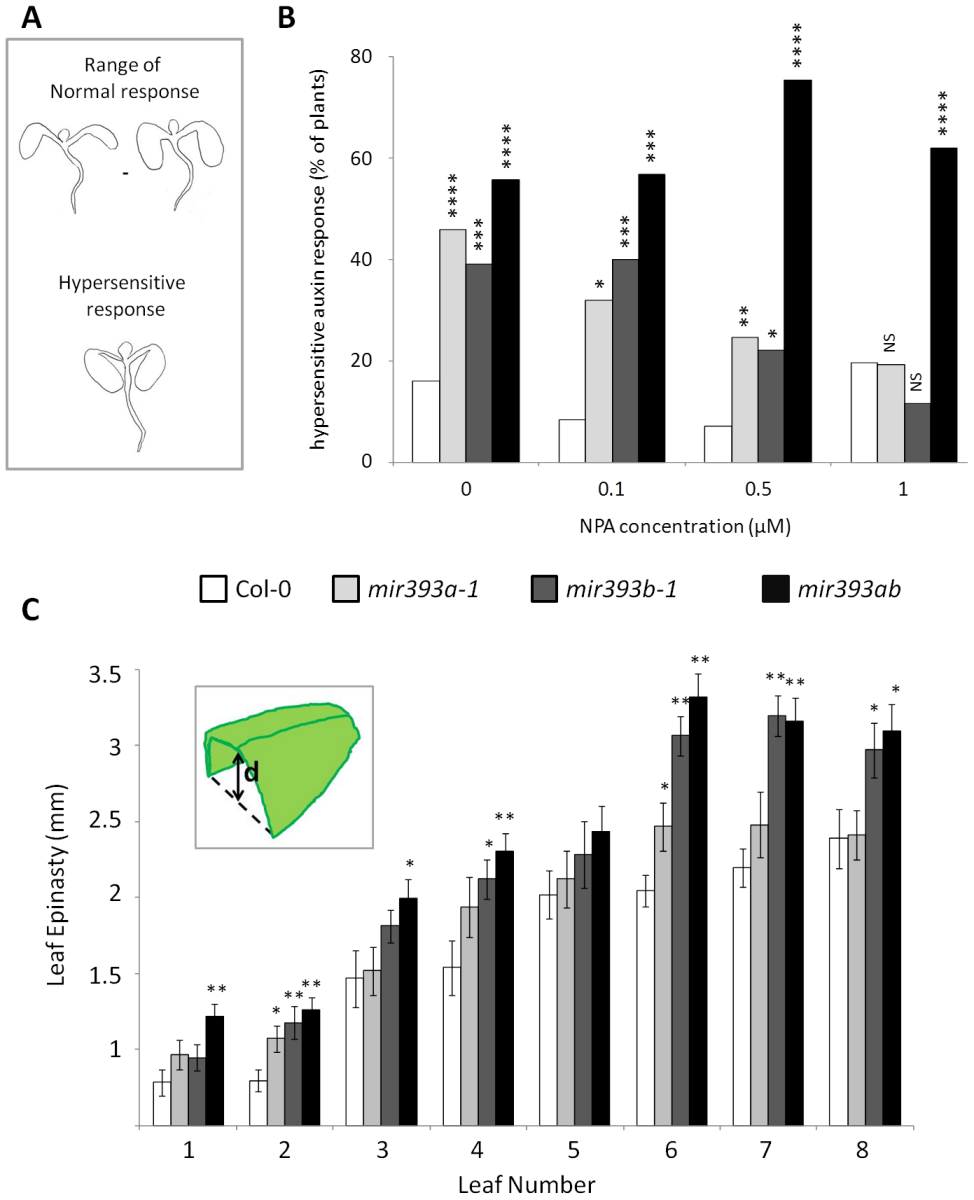
*AtMIR393A* and *AtMIR393B* are partially redundant for proper leaf morphogenesis. (A) Schematic representation of the normal range of cotyledon epinasty (top) and the extreme cotyledon epinasty (bottom) typical of the auxin-hypersensitive response. (B) The incidence of cotyledon auxin-hypersensitive response in populations of Col-0 (open bars), *mir393a-1* (light grey bars), *mir393b-1* (dark grey bars), and *mir393ab* double mutants (dark bars). Seedlings (n>40 for each condition and genotype) were grown on media containing the concentration of NPA indicated and harvested 4 d after germination. *P* values (two-tailed Fisher's exact test) for significant differences towards Col-0 are indicated; NS for *P*>0.05, * for *P*≤0.05, ** for *P*≤0.01, *** for *P*≤0.001, **** for *P*≤0.0001. *P* values for significant differences between mutants are given in Fig. S2. (C) Epinasty of leaf number 1 to 8 for Col-0, *mir393a-1*, *mir393b-1* and *mir393ab* was measured by the vertical distance between the adaxial leaf side and the leaf margin (in mm ± SEM) (see drawing in the insert). Significant difference towards Col-0 is indicated (two-tailed student *t*-test). * for *P*≤0.05, ** for *P*≤0.01. N = 10. *P* values for significant differences between mutants are given in Fig. S2.

A first inspection of the different mutant plants showed that neither *mir393a-1* nor *mir393ab* mutants exhibit a more drastic developmental defects than *mir393b-1*. We analyzed whether the two *MIR393* genes have redundant functions in leaf development by, first, recording the incidence of cotyledon epinasty (ICE) which is a typical auxin hypersensitive response regulated by miR393 [17], [22]. When grown on standard medium, a high and significantly greater fraction of *mir393a-1* (46%), of *mir393b-1* (39%) and of *mir393ab* mutants (56%) than wild-type plants (16%) exhibited the extreme cotyledon epinasty phenotype (Fig. 2A). In the cases of *mir393a-1* and *miR393b-1*, this ICE was similarly decreased by increasing the concentration of the auxin transport inhibitor NPA (1-N-naphthylphthalamic acid) to 0.1 or 0.5 µM and this ICE was similar to that of wt at a concentration of 1 µM (Fig. 2B) [23]. In *mir393ab* however the incidence of cotyledon epinasty remained significantly higher even at high NPA concentrations. These observations established that the cotyledon epinasty response to auxin depends on the overlapping function of both *MIR393* genes.

Next, we measured the degree of leaf epinasty for the first 8 leaves of the plants. For *mir393a-1* mutants, most of the leaves were more epinastic than wt plants but the difference to wt was significant only for leaves #2 and #6 (Fig. 2C). For *mir393b-1*, the epinasty was greater than that recorded for *mir393a-1* and was significantly different from wt for more than half of the leaves. For *mir393ab* mutants, the leaf epinasty was again greater than that of *mir393b-1* and the difference to wt was highly significant for all leaves except for leaf #5. These observations showed that *AtMIR393B* has a main role in the regulation of leaf epinasty and that *AtMIR393A* contributes in a slightly redundant manner to regulate the underlying developmental process.

### *MiR393*-Deficient Mutants Exhibit Decreased, rather than increased, Expression of *DR5pro:GUS*

The loss of miR393 and the consequent increase in *TAAR* genes' expression [10], [17] is expected to increase the degradation of AUX/IAA proteins and to increase the expression of *AUX/IAA* genes. However, because the expression of *AUX/IAA* genes is feedback-regulated by the AUX/IAA which they generate, it is uncertain at which level the homeostasis of *AUX/IAA* genes will then be maintained in miR393-deficient mutants. To gain insights into the role of miR393 in this homeostasis, we first analyzed the expression of the artificial auxin-induced gene *DR5pro:GUS*, which contains seven soybean auxin response elements (ARE) and serves to report the state of the auxin signalling output [24].

**Figure 3:**
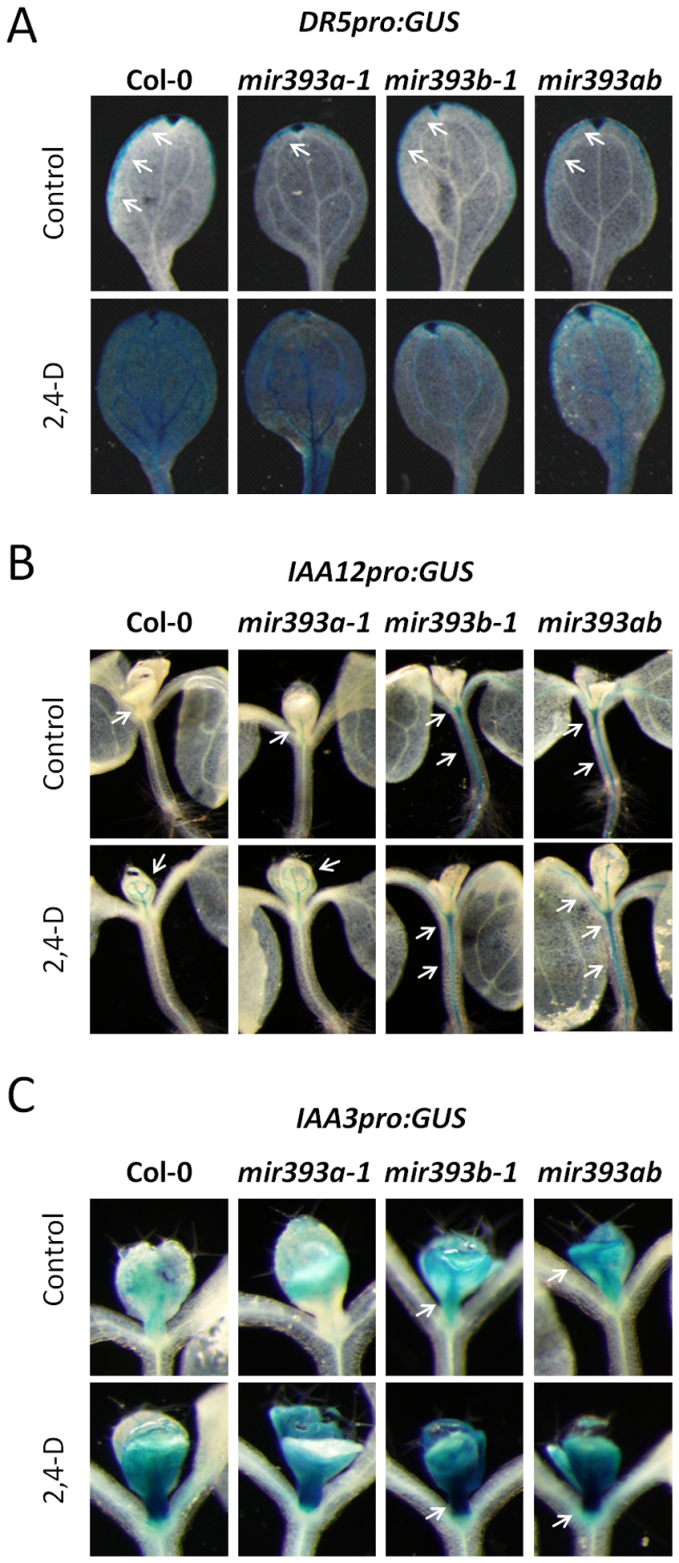
Basal expression level of primary auxin-inducible *AUX/IAA* genes in *mir393* mutants compared to wt plants. Pictures of representative wt and *mir393* mutant plants expressing the *DR5pro:GUS* (A), the *IAA12pro:GUS* (B), or the *IAA3pro:GUS* gene (C) upon treatment or not with 10 µM 2,4-D for 8 h. Arrows highlight the GUS staining detected.

Without treatments, the expression of *DR5pro:GUS* was similar in the distal tips of *mir393a-1*, *mir393b-1* and *mir393ab* and wt leaves while it was slightly lower in the margins of *mir393* mutants' leaves than in those of wt leaves (Fig. 3A). Treatment with 2,4-D, a synthetic diffusible auxin, induced the expression of *DR5pro:GUS* in all plant genotypes. However, its expression was lower in the blades of *mir393* mutants leaves, in the order *mir393a-1>mir393b-1 = mir393ab*, than in those of wt leaves (Fig. 3A). Thus, these results showed that the expression of *DR5pro:GUS* is changed in a counter-intuitive manner by the loss of miR393. *DR5pro:GUS* is more stably repressed in *mir393* mutants than in wt plants. Thus, these results suggest that the loss of miR393 leads to more complex changes than initially anticipated, especially at the level of AUX/IAA proteins.

### *MiR393*-Deficient Mutants Exhibit Increased Basal Expression of *AUX/IAA* Genes

To better understand the changes triggered by the loss of miR393, we analyzed the expression of artificial genes reporting the activity of the primary auxin-induced promoters of *IAA12/BDL and IAA3/SHY2* genes in wt and in miR393-deficient mutants (Fig. 3B–C). Without treatment, the expression level of *IAA12pro:GUS* was undetectable in wt plants, was weakly detectable in the shoot tips and roots of *mir393a-1* mutants, and was ectopically expressed at high levels in the shoot tips and hypocotyls of *mir393b-1* mutants (Fig. 3B). Interestingly, *IAA12pro:GUS* was also ectopically expressed in the shoot tips, in the hypocotyls and in the leaf veins of *mir393ab* mutants but at even higher levels. For *IAA3pro:GUS* we did not observe ectopic expression in the mutants but it was detected at high and increasing levels in the order *mir393b-1*<*mir393ab* while it was only weakly expressed in emerging leaves of wt and *mir393a-1* plants (Fig. 3C). Together these observations indicated that the two primary auxin-induced genes *IAA3/SHY2* and *IAA12/BDL* have higher basal expression levels in miR393-deficient mutants than in wt plants. Moreover, the mutation in *AtMIR393B* has a greater effect on *AUX/IAA* genes expression than the mutation in *AtMIR393A*.

Importantly, treatments of plantlets with 2,4-D for 8h induced the expression of *IAA12pro:GUS* and *IAA3pro:GUS* genes in all plant genotypes (Fig. 3B–C). In these conditions, the two reporter genes were also more expressed in *mir393a-1*, *mir393b-1* and *mir393ab* mutants than in wt plants. These data showed that miR393 is important to maintain the proper basal expression level of these two *AUX/IAA* genes and for their proper induction.

### *MiR393*-Deficient Mutants Exhibit Complex Changes in the steady state of *AUX/IAA* and *GH3* mRNAs

**Figure 4:**
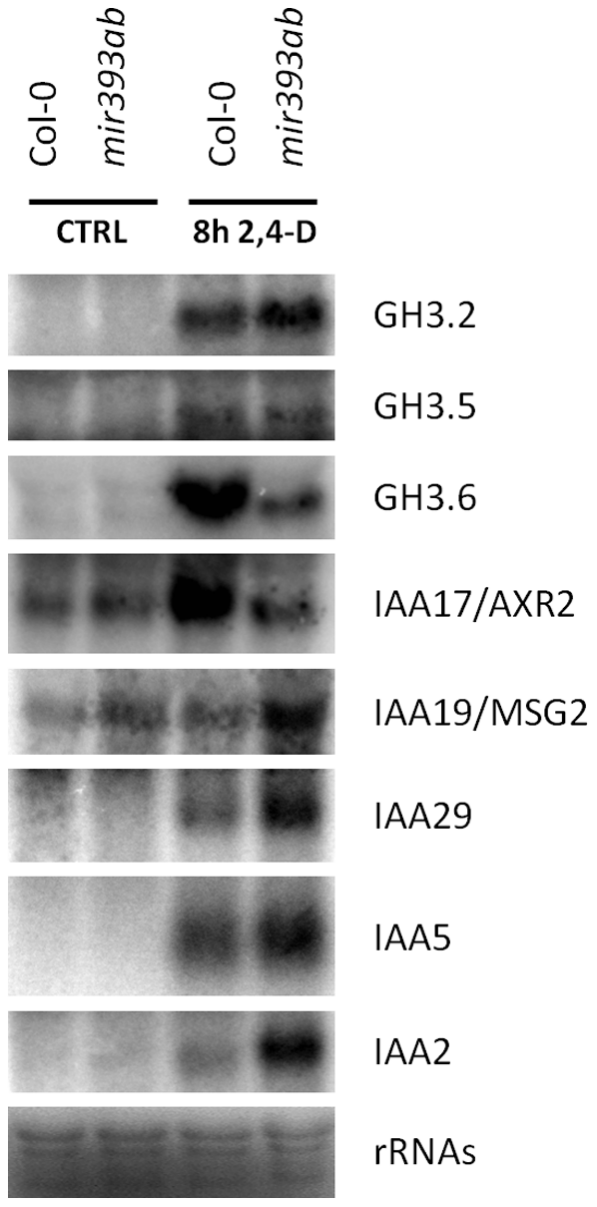
miR393 is required for proper induction of *AUX/IAA* and *GH3* genes. Northern blots of RNAs from wt and *mir393ab* mutant plantlets treated or not with 10 µM 2,4-D for 8 h. Without treatments, *IAA17* and *IAA19* have higher steady state levels in *mir393ab* mutants than in wt plants. Treatment with 2,4-D for 8 h induces the expression of all genes tested. *GH3.5* accumulates to similar levels in wt and mutants, *GH3.2*, *IAA19*, *IAA29*, *IAA5* and *IAA2* accumulate to higher levels in *mir393ab* mutants than in wt plants while *GH3.6* and *IAA17* accumulate to lower levels in *mir393ab* mutants than in wt plants.

To determine whether the observations made with the two reporter genes also apply to endogenous genes, we analyzed the mRNA steady state of few auxin-induced genes (Fig. 4). We selected 5 *AUX/IAA* and 3 *GH3* genes based on their high expression level reported in the *Arabidopsis eFP browser hormone series* (www.bar.utoronto.ca) [25]. Without auxin treatment, *IAA17* and *IAA19* mRNAs accumulated to higher levels in *mir393ab* mutants than in wt plants, and, the expression of the other 6 genes was not detected. The expression of all genes tested was induced by treatment with 2,4-D. In these conditions, *GH3.5* mRNAs accumulated to similar levels in wt and *mir393ab* mutants, *GH3.2*, *IAA19*, *IAA29*, *IAA5* and *IAA2* accumulated to higher levels in *mir393ab* mutants than in wt plants, while *GH3.6* and *IAA17* accumulated to lower levels in *mir393ab* mutants than in wt plants. Thus, although all genes tested were induced by auxin treatments in the *mir393ab* mutants, their accumulation was substantially different from that observed in wt plants. Together with the reporter genes analyses, these data showed that miR393 plays an important role to maintain the proper basal expression and for the accurate induction of *AUX/IAA and GH3* genes.

### *MIR393* is Necessary for Proper Degradation of *AXR3NT:GUS* Proteins

**Figure 5:**
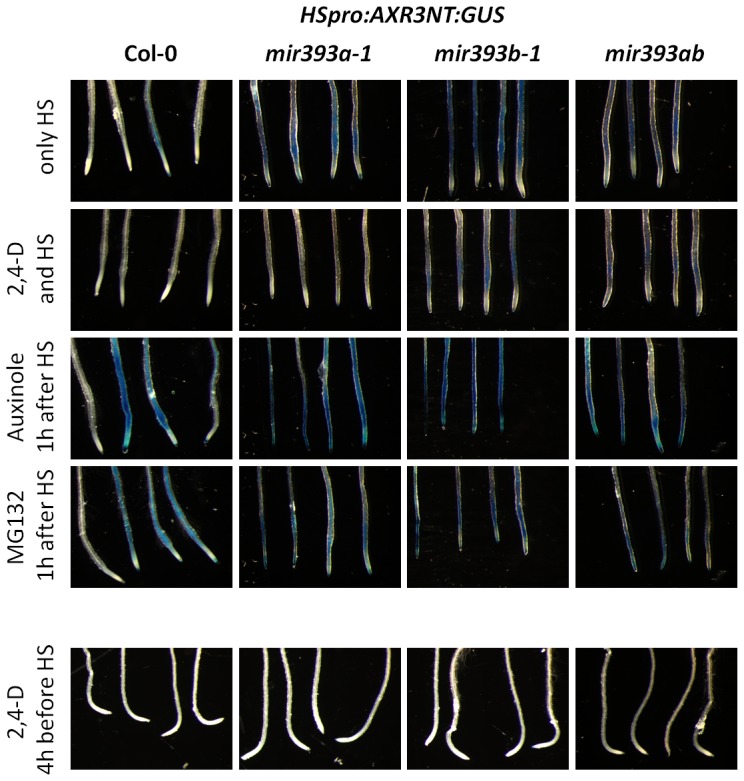
miR393 is required for proper degradation of AXR3-NT:GUS proteins. Representative pictures of wt and *mir393* mutant roots expressing the *HSpro:AXR3-NT:GUS* gene. Without treatments, the AXR3-NT:GUS fusion protein is more stable in *mir393a-1*, *mir393b-1* and *mir393ab* mutants compared to wt plants. Short treatments with 10 µM auxin induces the degradation of AXR3-NT:GUS, while treatment with 20 µM auxinole or MG132 blocks the degradation of AXR3-NT:GUS. This shows that the SCF complexes and proteasome are functional in the mutants as well. Clearance of presumably high levels of endogenous AUX/IAA proteins by treatment with 2,4-D for 4 h restores the proper degradation of AXR3-NT:GUS.

Next, we used the heat shock inducible *HSpro:AXR3-NT:GUS* line to assay the level of accumulation of AUX/IAA proteins and the efficiency of their degradation [7]. We anticipated that an increase in AUX/IAA protein steady-state levels should create a competition for ubiquitination and/or for degradation between AUX/IAAs and might therefore affect the degradation rate of AXR3-NT:GUS. After induction of the *HSpro:AXR3-NT:GUS* gene and without other treatments (Fig. 5; control) the level of AXR3-NT:GUS proteins was consistently higher in the roots of *mir393a-1*, *mir393b-1* and *mir393ab* mutants than in wt plants (first row). This result showed that in miR393-deficient mutants the AXR3-NT:GUS proteins are not degraded as efficiently as in wt plants. Brief treatment with 2,4-D during induction to enhance AUX/IAA protein degradation lead to a marked decrease in the accumulation of AXR3-NT:GUS proteins in *mir393* mutants compared to the control condition; Although the level was still higher in *mir393* mutants than in wt plants (second row). Similarly, brief treatment with auxinole (α-[2,4-dimethylphenylethyl-2-oxo]-IAA) which prevents the degradation of AUX/IAA proteins by binding to TIR1/AFBs [26] lead to increase the accumulation of AXR3-NT:GUS proteins (Fig. 5; third row). Brief treatment with MG132 which specifically inhibits the function of the proteasome didn't lead to substantial changes in the accumulation of AXR3-NT:GUS proteins (Fig. 5; fourth row). Thus, these experiments showed that the SCF^TIR/AFB^ complexes and the proteasome are functional, although less efficient, in *mir393* mutants than in wt plants. These experiments suggest that the level of auxins is the limiting factor and that the higher steady state level of endogenous AUX/IAA proteins in *mir393* mutants cannot be degraded as fast as in the wt. We further challenged this possibility by treating the plants with 2,4-D four hours before induction of the *HSpro:AXR3-NT:GUS* gene. As we anticipated, chasing the higher levels of AUX/IAA proteins by inducing their degradation with high quantities of auxin lead to restore the proper degradation of the AXR3-NT:GUS protein (Fig. 5; fifth row). Together these experiments showed that miR393 is required to ensure the proper degradation of the AXR3-NT:GUS fusion proteins and of other AUX/IAA proteins. Moreover, miR393-deficient mutants seem to indeed accumulate higher steady state levels of endogenous AUX/IAA proteins.

## Discussion

Auxins are major plant hormones with crucial functions in almost every aspect of plant life [1], [2]. Identification of mutants affected in synthesis, transport and perception of auxins have demonstrated the importance of auxins in developmental and morphogenetic programs [5], [27]. MiR393 is encoded by two distinct genes and regulates the expression of *TIR1/AFB2* auxin receptor genes. According to the crucial role of auxins, we anticipated that miR393-deficient mutants, which we showed in *mir393b-1* mutants, fail to regulate *TAAR* mRNA levels, should exhibit pleiotropic developmental defects. However, no obvious developmental defects other than mild leaf polarity defects were observed in the single mutants *mir393a-1*, *mir393b-1* or in the double mutants *mir393ab* grown in standard laboratory conditions. We suspect that *mir393a-1* is not a null mutant since this mutation leads to a 30% decrease in the level of mature miR393 in the roots. Another possibility is that a third unidentified *MIR393* gene produces the remaining miR393 in the roots. Chen and colleagues have shown that the expression of *MIR393* and *TAAR* genes are under transcriptional feedback regulations [19]. Thus, it is possible that these feedbacks can compensate for the loss of miR393. However, we do not favor this possibility since we have observed clear effects on expression of primary auxin-induced genes and on degradation of AUX/IAA proteins in miR393-deficient mutants. Thus, we suspect that other unrelated pathways, rather than the above mentioned feedback mechanisms solely, act redundantly with miR393 and somehow compensate for its loss.

Although *mir393a-1* is unlikely a null mutant, our studies with *mir393a-1*, *mir393b-1* and *mir393ab* has shown that both *MIR393* genes contribute in a partially redundant fashion to the establishment of leaf polarity in arabidopsis; with *AtMIR393B* gene playing the predominant role. We found that all miR393-deficient mutants exhibit a hypersensitive auxin response characterized by an extreme epinasty of the cotyledons. For *mir393a-1* and *mir393b-1*, the hypersensitivity phenotype was prevented when the plants were grown on increasing concentrations of the auxin transport inhibitor NPA. For *mir393ab* double mutants the extreme auxin response was not prevented by the highest concentrations of NPA. When we increased the concentration of NPA to 2 µM or higher, the cotyledon epinasty phenotype could not be analyzed appropriately since the plants did not germinate or grow correctly (not shown).

Auxin homeostasis relies on intricate feedback regulations at several levels including synthesis, transport and signaling *via* AUX/IAA proteins. The data which we report here add to the current picture and show that the regulation of *TAAR* genes homeostasis by miR393 plays a significant role for the homeostasis of auxin signaling. We found that the basal steady-state expression level of *IAA12pro:GUS*, of *IAA3pro:GUS* and of several endogenous *AUX/IAA* genes is higher in miR393-deficient mutants than in wt plants. Importantly, this and additional experiments suggest that the increase in *AUX/IAA* gene expression leads to a concomitant increase in the basal steady state level of AUX/IAA proteins. These observations are counter-intuitive on first sight since an increase in the expression level of *AUX/IAA* genes leading to an increase in the level of AUX/IAA proteins should result in a decrease in the expression of *AUX/IAA* genes. Conversely, a decrease in the expression level of *AUX/IAA* genes leading to a decrease in the level of AUX/IAA proteins should result in an increase in the expression of *AUX/IAA* genes. We believe that the counter-intuitive observations are due to the intrinsic homeostatic nature of *AUX/IAA* genes which are feedback regulated by the AUX/IAA proteins which they generate. Indeed, such feedback-regulated systems lead to meta-stable steady state levels. Thus, we believe that the *AUX/IAA* genes, which exhibit a concomitant increase in gene expression and in the level of the corresponding AUX/IAA proteins, have actually reach a higher meta-stable steady-state level in miR393-deficient mutants than in wt plants. We have not been able to challenge this hypothesis by straightforward measurements of endogenous AUX/IAA protein levels because antibodies for AUX/IAA proteins or arabidopsis lines expressing tagged versions of AUX/IAA proteins under their native promoters are not available. We speculate that the expression of a given *AUX/IAA* gene should be even higher in a *mir393ab* mutant expressing non functional AUX/IAA proteins. However, this approach would require the production of mutants with several orders of magnitude. However, we have been able to provide indirect evidences supporting our hypothesis. Indeed, experiments using *HSpro:AXR3-NT:GUS* showed that the AXR3-NT:GUS fusion proteins are not properly degraded in miR393-deficient mutants. This indicated that the proper degradation of the AXR3-NT:GUS proteins in auxin-limiting conditions is prevented by the high levels of AUX/IAA proteins. This conclusion was also supported by the observations that treatment of plants with 2,4-D for 4 h before inducing the *HSpro:AXR3-NT:GUS* gene, which induces the degradation of endogenous AUX/IAA proteins, could restore the proper degradation of AXR3-NT:GUS. Finally, expression analysis of *DR5pro:GUS*, the universal auxin signalling output marker gene, also support our hypothesis since its expression level was slightly lower in miR393-deficient mutants than in wild type plants, and thus indicate that it is slightly more repressed in mR393-deficient mutants than in wt plants.

The delay of AXR3-NT:GUS degradation is more pronounced in the roots of *mir393b-1* than in those of *mir393a-1*. However, our sRNA blot experiments showed than miR393 accumulates to lower levels in the roots of *mir393a-1* than in those of *mir393b-1*. Thus, this discrepancy suggests that the loss of miR393 observed in the shoots of *mir393b-1* leads to increase the level of competing AUX/IAA proteins in the roots. We speculate that this is achieved either by an increased synthesis or by an increased transport.

Earlier studies had use miR393-resistant target genes and miR393 overexpressers to unravel the function and roles of miR393 [10]-[12], [14], [15], [19]. However, although miR393 had been shown to regulate the expression of *TAAR* genes and of auxin-responsive genes, and moreover to be involved in important biological processes, its functional significance for auxin signalling and its homeostasis had not really been evaluated directly. Our experiments now clarify the picture and demonstrate that miR393 is necessary to maintain low basal expression levels of *AUX/IAA* genes, low basal levels of AUX/IAA proteins and that these features are important for the proper degradation of AUX/IAA proteins. Thus, miR393 is not only important for *TAAR* genes homeostasis but also for the establishment of proper auxin signalling outputs and for auxin signalling homeostasis *per se*.

## Materials and Methods

### Plant Material

*miR393a-1* mutant was obtained by PCR-based genotyping of plants from the seed batch WiscDsLoxHS224_12B obtained from the NASC stock center. The *mir393ab* double mutant was obtained by PCR-based genotyping of F2 seedlings obtained from the cross of *mir393a-1* and *mir393b-1* [17]. The *DR5pro:GUS*, *IAA12pro:GUS*, *IAA3pro:GUS* and *HSpro:AXR3-NT:GUS* reporters were introgressed in *mir393a-1*, *mir393b-1* and *mir393ab* mutants by crossing and homozygous plants were found by PCR-based genotyping in the F2 population. In all cases, double homozygosis was ascertained on at least 9 plants of the F3 population.

### Growth Conditions & Treatments

*Arabidopsis thaliana* plants used to prepare RNA blots and to measure leaf epinasty were grown on soil in the greenhouse as described previously [28].

Studies of cotyledon epinasty were done as we previously described [17] on Murashige and Skoog (MS) solid medium supplemented as indicated with NPA (1-N-naphthylphthalamic acid, Fluka) in DMSO or DMSO alone as a control.

Measurements of leaf epinasty were made on plants grown for 45 days in short-day (SD) conditions (8 h light/16 h dark). Root measurements were made on plantlets germinated on MS medium and transferred for 8 days in vertically oriented square-plates on solid MS medium supplemented as indicated with 0.1 µM 2,4-D or 20 µM auxinole in SD conditions. For studies of *IAA3pro:GUS*, *IAA12pro:GUS* and *DR5pro:GUS* expression, 7 days old seedlings were incubated in MS medium containing 10 µM 2,4-D in ethanol or ethanol alone as a control for the time indicated before proceeding to staining. For studies of AXR3-NT:GUS fusion protein stability, 7 days old seedlings were placed in water at 37°C for 2 h to induce the expression of the *HSpro:AXR3-NT:GUS* gene and incubated for 30 minutes at room temperature before proceeding to GUS staining. For the treatments, either 10 µM 2,4-D were added to the solution just before the heat shock or 20 µM auxinole were added after 1 h of heat-shock as it was described [7], [26].

### RNA preparation and RNA Analysis

Extraction of total RNAs, preparation of RNA blots and qRT-PCRs were done as previously described [28].

### Histochemical GUS Assays

Arabidopsis seedlings were incubated into staining solution containing 1 mM X-Gluc in 100 mM Na3PO4 (pH 7.2), 0.1% Triton X-100, 5 mM K3Fe(CN)6 and 5 mM K4Fe(CN)6 for 24 h at 37°C in the dark [29]. Seedlings were then cleared in 70% ethanol for 2 days and mounted in 50% v/v glycerol before observations.

## Supporting Information

Figure S1 **Position of** ***miR393a-1*** **T-DNA insertion in** ***AtMIR393A*** **(At2g39885).** The pri-miRNA sequence (546-nt) which we identified by RACE experiments is indicated in orange color. The pre-miRNA sequence (133-nt) is in typed in capital letters and the miR393 sequence (22-nt) is underlined. The T-DNA insertion is located between the two nucleotides highlighted in red.
(DOCX)

Figure S2 ***AtMIR393A*** **and** ***AtMIR393B*** **are partially redundant for proper leaf morphogenesis.** (A–B) The incidence of cotyledon auxin-hypersensitive response in populations of Col-0 (open bars), *mir393a-1* (light grey bars), *mir393b-1* (dark grey bars), and *mir393ab* double mutants (dark bars). Seedlings (n>40 for each condition and genotype) were grown on media containing the concentration of NPA indicated and harvested 4 d after germination. *P* values (two-tailed Fisher's exact test) for significant differences towards *mir393a-1* (A) or *mir393b-1* (B) are indicated; NS for *P*>0.05, * for *P*≤0.05, ** for *P*≤0.01, *** for *P*≤0.001, **** for *P*≤0.0001. (C–D) Epinasty of leaf number 1 to 8 for Col-0, *mir393a-1*, *mir393b-1* and *mir393ab* was measured by the vertical distance between the adaxial leaf side and the leaf margin (in mm ± SEM). Significant difference towards *mir393a-1* (C) or *mir393b-1* (D) are indicated (two-tailed student *t*-test). * for *P*≤0.05, ** for *P*≤0.01. N = 10.
(TIF)
