# Supplementary material for: miR393 Is Required for Production of Proper Auxin Signalling Outputs
Source: PLoS One. 2014 Apr 24;9(4):e95972. doi: 10.1371/journal.pone.0095972 (PMC3999107; doi:10.1371/journal.pone.0095972)
Supplement: Figure S1 — Position of miR393a-1 T-DNA insertion in AtMIR393A (At2g39885). The pri-miRNA sequence (546-nt) which we identified by RACE experiments is indicated in orange color. The pre-miRNA sequence (133-nt) is in typed in capital letters and the miR393 sequence (22-nt) is underlined. The T-DNA insertion is located between the two nucleotides highlighted in red. (DOCX) [file pone.0095972.s001.docx]

**Fig. S1. Position of *miR393a-1* T-DNA insertion in *AtMIR393A* (At2g39885)**

The pri-miRNA sequence (546-nt) which we identified by RACE experiments is indicated in orange color. The pre-miRNA sequence (133-nt) is in typed in capital letters and the miR393 sequence (22-nt) is underlined. The T-DNA insertion is located between the two nucleotides highlighted in red.

ttaaaataagccaacaaaatttagtaaagacgaaaataaataatagtgtcacccaacttgtggggaatatatatctaaattttgctgtgatagagcgtgt

tttgtttgagtagttgatttctcaagtaaatcacttgctttatagtaaaagagaaaaacatttagtcattttgacctactacgtacccatcatgaacact

gtgttgcaatttttaagagtccattaagaaaattacaattttgtgctcatctatgcatgtgtcaaccgcaaaatcatcatataattttactagctacagt

ctcatcaccatataaccactaatccgtttatacttattaagagcccatacaaaaatttgtacagaaacgtagacgtctggtttactagctccataagtca

atataaaaatggaaaaccaaaagttggaaaataatataaaaataaataaataaatttgaagggtcagaaagtggaaactaaaagataaatgagtattatt

taaaatcaagaggaacacgatccattgacaaaaaccacattgctctcaacttttagagtgagagagagatagagagttgaacaaattcttcatagcaact

AGAGGAAGGATCCAAAGGGATCGCATTGATCCTAATTAAGGTGAATTCTCCCCATATTTTCTTTATAATTGGCAAATAAATCACAAAAATTTGCTTGGTT

TTGGATCATGCTATCTCTTTGGATTCATCCTTCggtagcttcctcttcgatcgtatggcaaatttctcaactatcatggatacttcaccgggaactttgc

tggctttaagattccgcaaccttgtatctcaagcctacaacagtatacgaactctcttcttacaattttcattttttaaaaggtaagtttcaaagaattt

tgacctaatcatgcaaaaccaaaaaagaagaagaagtcacctacctgtttttttttggctttcttcttcattggattttgagatcgaatttccaattaaa

aattagcttatgaaatttatatattttatgaaagacgtcattttaaagttagtttaaaatttctctgaaata
