# Supplementary figures and images for: miR393 Is Required for Production of Proper Auxin Signalling Outputs
Source: PLoS One. 2014 Apr 24;9(4):e95972. doi: 10.1371/journal.pone.0095972 (PMC3999107; doi:10.1371/journal.pone.0095972)

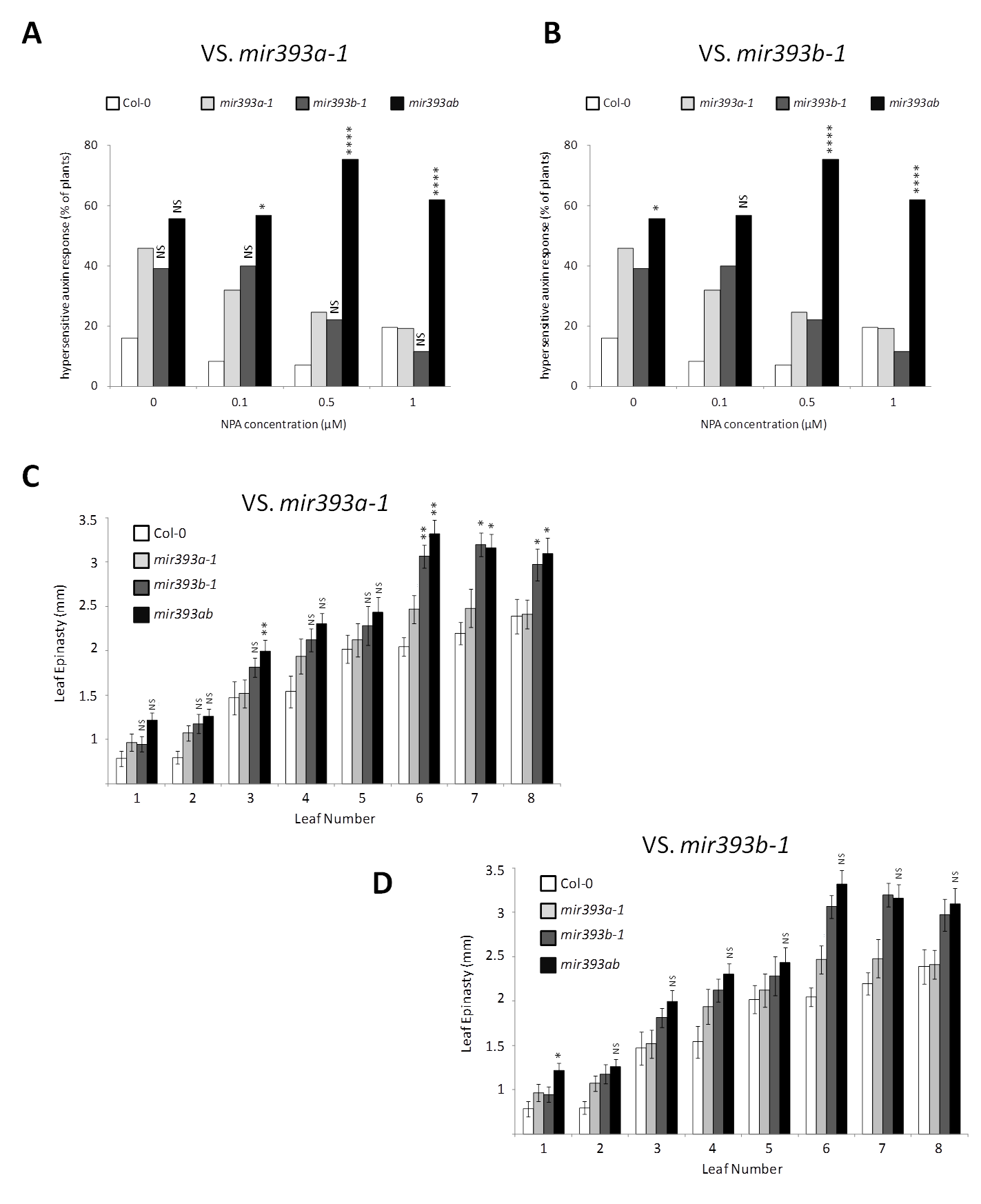

Supplement: Figure S2 — AtMIR393A and AtMIR393B are partially redundant for proper leaf morphogenesis. (A–B) The incidence of cotyledon auxin-hypersensitive response in populations of Col-0 (open bars), mir393a-1 (light grey bars), mir393b-1 (dark grey bars), and mir393ab double mutants (dark bars). Seedlings (n>40 for each condition and genotype) were grown on media containing the concentration of NPA indicated and harvested 4 d after germination. P values (two-tailed Fisher's exact test) for significant differences towards mir393a-1 (A) or mir393b-1 (B) are indicated; NS for P>0.05, * for P≤0.05, ** for P≤0.01, *** for P≤0.001, **** for P≤0.0001. (C–D) Epinasty of leaf number 1 to 8 for Col-0, mir393a-1, mir393b-1 and mir393ab was measured by the vertical distance between the adaxial leaf side and the leaf margin (in mm ± SEM). Significant difference towards mir393a-1 (C) or mir393b-1 (D) are indicated (two-tailed student t-test). * for P≤0.05, ** for P≤0.01. N = 10. (TIF) [file pone.0095972.s002.tif]
